# HDL structure and function is profoundly affected when stored frozen in the absence of cryoprotectants[S]

**DOI:** 10.1194/jlr.D075366

**Published:** 2017-09-11

**Authors:** Michael Holzer, Sabine Kern, Markus Trieb, Athina Trakaki, Gunther Marsche

**Affiliations:** Institute of Experimental and Clinical Pharmacology,* Medical University of Graz, Graz, Austria; BioTechMed-Graz,† Graz, Austria

**Keywords:** high density lipoprotein, paraoxonase, cholesterol efflux capacity, apolipoproteins, high density lipoprotein/structure, long-term storage, freezing, glycerol, sucrose

## Abstract

Analysis of structural and functional parameters of HDL has gained significant momentum in recent years because they are stronger predictors of cardiovascular risk than HDL-cholesterol levels. Surprisingly, in most HDL studies, very low attention is paid to HDL storage, which might critically affect functional properties. In the present study, we systematically examined the impact of storage and freezing on the structural/functional properties of freshly isolated HDL. Initial damage to HDL starts between week 1 and week 4 of storage. We observed that prolonged freezing at −20°C or −70°C led to a shedding of apoA-I from HDL and to the formation of large protein-poor particles, indicating that HDL is irreversibly disrupted. These structural alterations profoundly affected key metrics of HDL function, including HDL-cholesterol efflux capacity and HDL paraoxonase activity. Flash-freezing of isolated HDL prior to storage at −70°C did not preserve HDL structure. However, addition of the cryoprotectants, sucrose or glycerol, completely preserved structure and function of HDL when stored for at least 2 years. Our data clearly indicate that HDL is a complex particle requiring special attention when stored. Addition of cryoprotectants to isolated HDL samples before storage will make biochemical and clinical HDL research studies more reproducible and comparable.

Epidemiological studies have shown that serum levels of HDL-cholesterol have a strong inverse correlation with cardiovascular disease risk in the general population (1). The protective effect of HDL has been classically attributed to the amount of HDL present and its ability to promote reverse cholesterol transport, a series of processes by which HDL is able to transport cholesterol from the periphery back to the liver for excretion (2). In addition to its role in cholesterol metabolism, HDL inhibits oxidative and inflammatory processes and modulates functions of a variety of cell types, including macrophages, neutrophils, and endothelial cells (3–8). Over the past decade, the concept emerged that HDL can become dysfunctional, thereby losing its protective activities. Such dysfunctional forms of HDL are present in patients suffering from coronary artery disease, myocardial infarction, end stage renal disease, diabetes, arthritis, psoriasis, and other metabolic or inflammatory diseases (9–18). These observations have spurred numerous studies to investigate the functional properties of HDL in chronic diseases and the effect of therapeutics on HDL composition and function. Most studies used isolated HDL to test the functional properties of HDL directly, while others used apoB-depleted serum as a specimen to predict HDL function. However, while a lot of effort has been made to develop assays to study the metrics of HDL function, not much attention has been given to the impact of storage conditions of the HDL used and the serum it was derived from. As early as 1946, it was described that the attachment of proteins and lipids within lipoproteins appeared to be weakened by freezing (19). Storage of human serum at −20°C or −70°C has been found to change the recovery of individual lipoprotein fractions upon ultracentrifugation or precipitation, although the total cholesterol and triglyceride content was not altered (20–22). Studies on HDL-cholesterol levels in serum have shown that the concentration of measureable HDL-cholesterol after freezing can decrease by a rate of up to 12% per year depending on the storage condition (23–25). Thus, long-term storage of plasma seems to be unsuitable for the later quantification and isolation of HDL. These observations suggest that separation of lipoprotein fractions, including HDL, from serum should, ideally, be performed as soon as possible after blood collection. However, this will also require that HDL, upon isolation, can be stored for an extended period. However, only few data are available about the impact of long-term storage conditions on HDL structure and function. Therefore, our study aims to explore the impact of storage conditions and the benefit of using cryoprotectants for long-term storage of isolated HDL on its molecular structure and its functional properties.

## MATERIALS AND METHODS

### Blood collection and isolation of HDL

Blood was sampled from 12 healthy volunteers after obtaining written informed consent. The study protocol and all study procedures were reviewed and approved by the local ethics committee (Number 21-523 ex 09/10). All participants gave written informed consent before being enrolled into the study. Serum from four healthy controls was pooled for HDL isolation and storage experiments were repeated three times. Serum density was adjusted with potassium bromide (Sigma, Vienna, Austria) to 1.24 g/ml and a two-step density gradient was generated in centrifuge tubes (16 × 76 mm; Beckman) by layering the density-adjusted plasma (1.24 g/ml) underneath a NaCl-density solution (1.006 g/ml), as described (26). Tubes were sealed and centrifuged at 415,000 *g* for 6 h in a 90Ti fixed angle rotor (Beckman Instruments, Krefeld, Germany). After centrifugation, the HDL-containing band was collected, desalted via PD10 columns (GE Healthcare, Vienna, Austria), and immediately used for experiments.

### Storage experiments with isolated HDL

For each experiment, individual aliquots of isolated HDL were prepared and used only once. Isolated HDL was either stored at 4°C or frozen at −20°C or −70°C. For flash freezing experiments, freshly isolated HDL samples were flash-frozen in liquid nitrogen for 5 min and afterwards transferred into a −70°C freezer. Glycerol (10–50%) or sucrose (2–10%) was added directly before freezing.

### Native gel electrophoresis

Isolated HDL (5–20 μg protein per lane) was separated by gradient gel electrophoresis (4–16% NativePage; Life Technologies, Vienna, Austria) under nonreducing and nondenaturing conditions. Afterwards, gels were either stained for proteins with a freshly prepared solution of Coomassie Brilliant Blue G-250 overnight (Thermo Scientific, Rockford, IL) or stained for lipids using Sudan black. To estimate the size of isolated HDL, we used a protein standard containing BSA (66 kDa, 7.1 nm), lactate dehydrogenase (146 kDa, 8.2 nm), B-phycoerythrin (242 kDa, 10.5 nm), apoferritin band 1 (480 kDa, 12.2 nm), and apoferritin band 2 (720 kDa, 18.0 nm) (NativeMark, Life Technologies).

### Immunoblotting

For blotting, gels were transferred to PVDF membranes with 100 V for 60 or 90 min at 4°C for native gels or SDS-PAGE, respectively. Membranes were probed with the following primary antibodies diluted in 5% milk overnight at 4°C: apoA-I antibody (Nr.: ab64308, 1:1,000 dilution; Abcam), apoA-II (Nr.: ab92478, 1:1,000 dilution; Abcam), apoC-I (Nr.: BP2081, 1:1,000 dilution; Acris), serum amyloid A (SAA) (Nr.: RAS-H-SAA-A8, 1:1,000 dilution; courtesy of G. Kostner, Graz, Austria). Membranes were washed and incubated with secondary HRP-conjugated antibodies for 2 h at ambient temperature. Membranes were carefully washed and developed using reagents and detection was performed on a Chemidoc Touch imaging system (Bio-Rad, Vienna, Austria).

### Cholesterol efflux capability of isolated HDL

Cholesterol efflux capacity was assessed using an established assay (27). J774 macrophages, maintained in DMEM with 10% fetal bovine serum, 1% penicillin-streptomycin, were plated on 48-well plates (300,000 cells/well). Cells were labeled for 24 h with 1 μCi/ml [^3^H]cholesterol (Perkin Elmer, Boston, MA). To upregulate ABCA1, cells were stimulated for 6 h with serum-free DMEM, 1% penicillin-streptomycin, containing 0.3 mmol/l 8-(4-chlorophenylthio)-cyclic AMP (Sigma, Darmstadt, Germany). After labeling, cells were rinsed and [^3^H]cholesterol efflux was determined by incubating the cells for 4 h with 2.8% apoB-depleted serum or 50 μg/mg HDL protein. Cholesterol efflux capacity is expressed as radioactivity in the medium relative to total radioactivity in medium and cells. All steps were performed in the presence of 2 μg/ml of the acyl-CoA cholesterol acyltransferase inhibitor, Sandoz 58-035 (Sigma).

### Arylesterase activity assay

Ca^2+^-dependent arylesterase activity was determined using a photometric assay with phenylacetate as substrate. HDL (0.5 μg protein) was added to 200 μl buffer containing 100 mmol/l Tris, 2 mmol/l CaCl_2_ (pH 8.0), and phenylacetate (1 mmol/l). The rate of hydrolysis of phenylacetate was monitored by the increase of absorbance at 270 nm and readings were taken every 30 s at room temperature to generate a kinetic plot. The slope from the kinetic chart was used to determine ΔAb270 nm/min. Enzymatic activity was calculated with the Beer-Lambert Law from the molar extinction coefficient of M^−1^ × cm^−1^ for phenylacetate.

### Statistical analysis

Comparisons of multiple groups were done with one-way ANOVA and Dunnett’s post hoc test. Significance was accepted at *P* < 0.05. Statistical analyses were performed with GraphPad Prism version 5.

## RESULTS

**Fig. 1.**
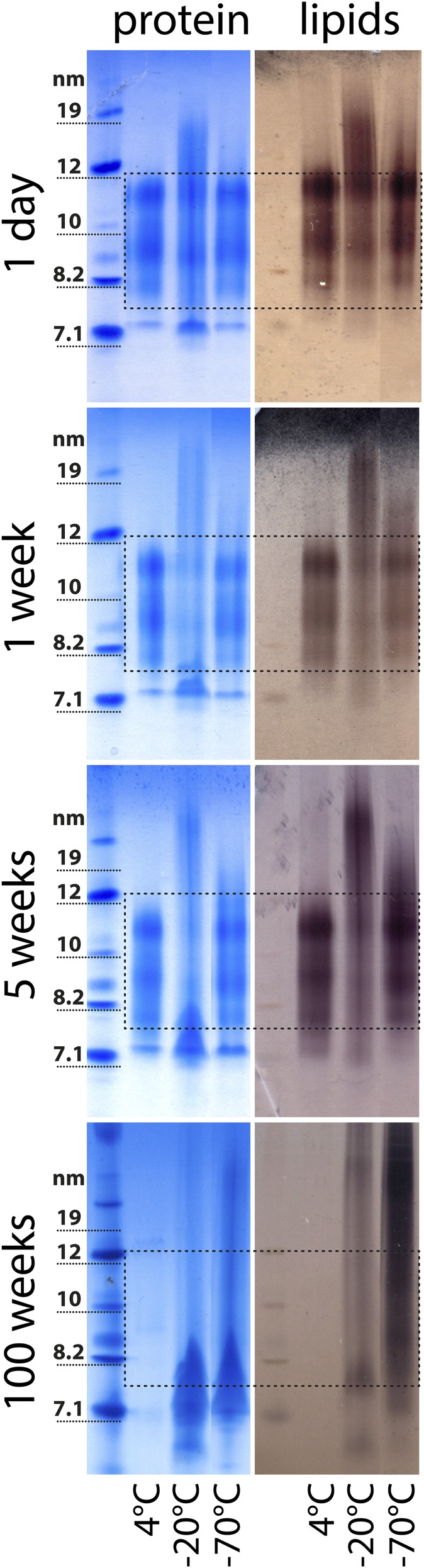
Native gel electrophoresis of isolated HDL after short- and long-term storage. HDL was isolated from serum from four healthy donors by density gradient ultracentrifugation and kept at 4, −20, or −70°C for up to 100 weeks. Aliquots were analyzed by native gel electrophoresis at different time points and stained with Coomassie brilliant blue for protein (left panel) or with Sudan black B for lipids (right panel). The dashed lines indicate the typical size range of HDL_2_ and HDL_3_ ranging from about 8 to 12 nm. HDL samples shown were from one gel, but were not directly adjacent to each other and, therefore, have been cut and assembled for illustration. Each storage experiment was repeated three times and results from one representative experiment are shown.

To investigate the impact of different storage conditions on the molecular integrity of HDL, we stored HDL at 4, −20, or −70°C for up to 100 weeks. Samples analyzed at different time points showed that freezing caused marked structural alterations (**Fig. 1**). These structural changes became more pronounced over the tested storage periods and led to shedding of proteins from HDL. The loss of proteins resulted in the formation of lipid-poor apoA-I and large protein-poor lipid particles (Fig. 1). These observations indicate that HDL is not stable when kept frozen at −20°C or −70°C for extended periods of time. Initial damage to frozen HDL started between week 2 and week 4 of storage, dependent on the individual isolation (**Figs. 1**, ** 2**). Alternatively, HDL can be stored for a short-term at 4°C. We observed that, at 4°C, HDL was stable for up to 2 weeks (Fig. 1, supplemental Fig. S1).

**Fig. 2.**
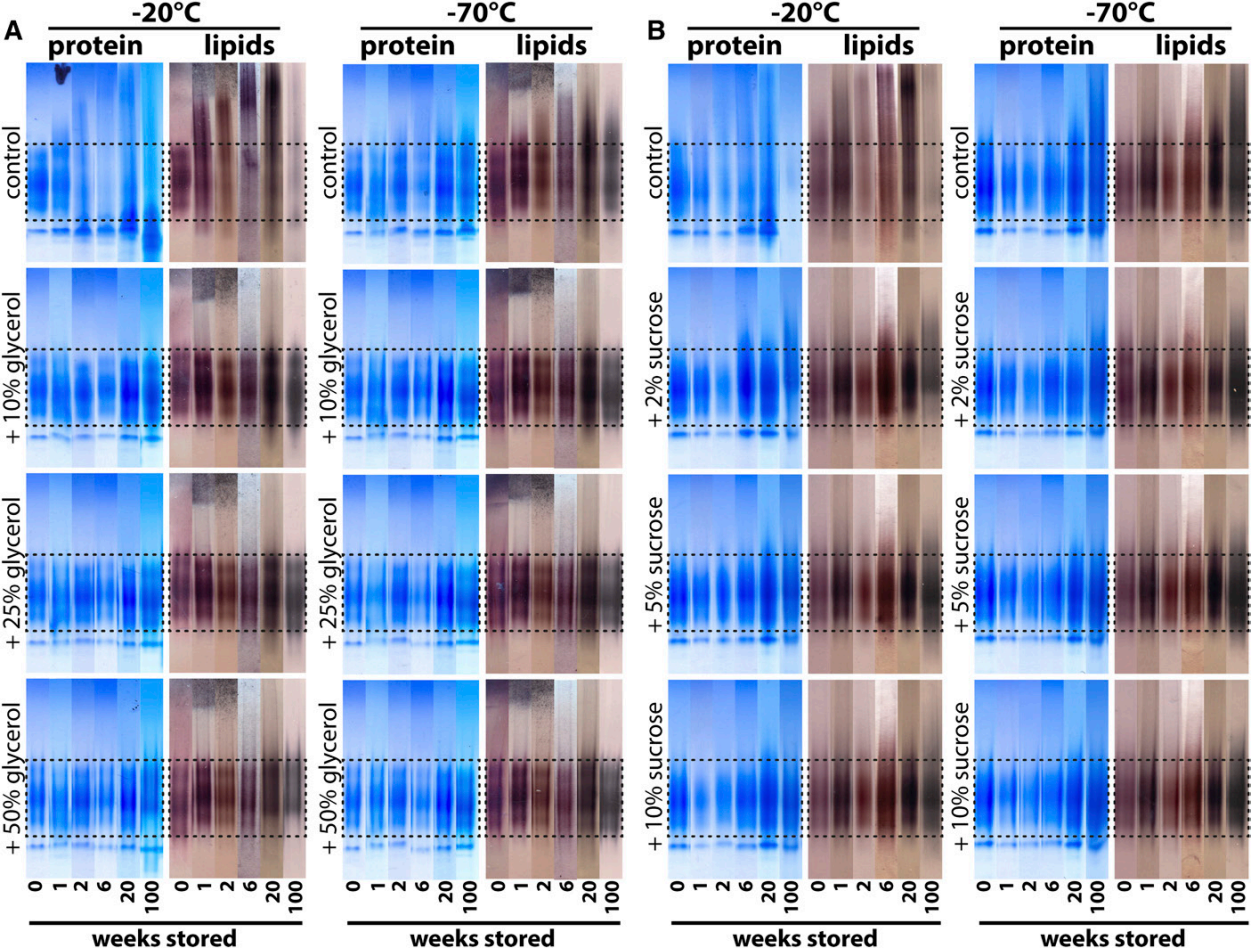
Native gel electrophoresis of cryopreserved HDL. HDL isolated by density gradient ultracentrifugation was stored at −20°C or −70°C in the presence or absence of the cryoprotectants glycerol (10–50%) (A) or sucrose (2–10%) (B) for up to 100 weeks. Aliquots were analyzed at different time points and stained with Coomassie brilliant blue for protein or with Sudan black B for lipids. The dashed lines indicate the typical size range of HDL_2_ and HDL_3_ ranging from about 8 to 12 nm. To be able to illustrate structural changes of HDL upon long-term storage, we have placed the analyzed time points from each sample next to each other. Samples represent pooled HDL preparation from four healthy donors. Each storage experiment was repeated three times and results from one representative experiment are shown.

**Fig. 3.**
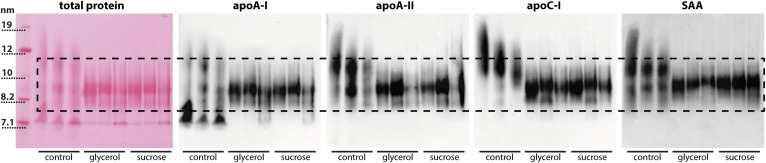
Immunoblot analysis of HDL stored with and without cryoprotectants. HDL was isolated from serum by density gradient ultracentrifugation. HDL was stored at −20°C or −70°C in the presence or absence of 25% glycerol or 5% sucrose for 25 weeks. Three samples for each condition (control, glycerol, or sucrose) from three individual donors are shown. Proteins were separated by native gel electrophoresis and blotted onto PVDF membranes. Blots were probed using antibodies for apoA-I, apoA-II, apoC-I, and SAA. Total protein was visualized after blotting by Ponceau red staining. The dashed line indicates the typical size range of HDL_2_ and HDL_3_ ranging from about 8 to 12 nm.

We next tested to determine whether the cryoprotectants sucrose and glycerol are able to maintain HDL’s molecular integrity. HDL was stored with either 2–10% sucrose or with 10–50% glycerol at −20°C or −70°C for up to 100 weeks (Fig. 2). The control preparations, without the addition of cryoprotectants, showed the same structural alterations, as shown in Fig. 1, starting from week 1. However, upon the addition of at least 5% sucrose or 10% glycerol, the molecular integrity of HDL was completely preserved (Fig. 2). Notably, low concentrations of sucrose (2%) at −20°C did not completely prevent storage-induced damage to HDL, suggesting that at least 5% of sucrose is needed for long-term storage of HDL (Fig. 2). We used immuno­blot analysis to test which main HDL proteins are affected by different storage conditions of HDL. Our analysis suggests that primarily apoA-I is shed from mature HDL, while other major HDL proteins, such as apoA-II, apoC-I, and SAA, appear to dissociate to a much lower extent (**Fig. 3**).

**Fig. 4.**
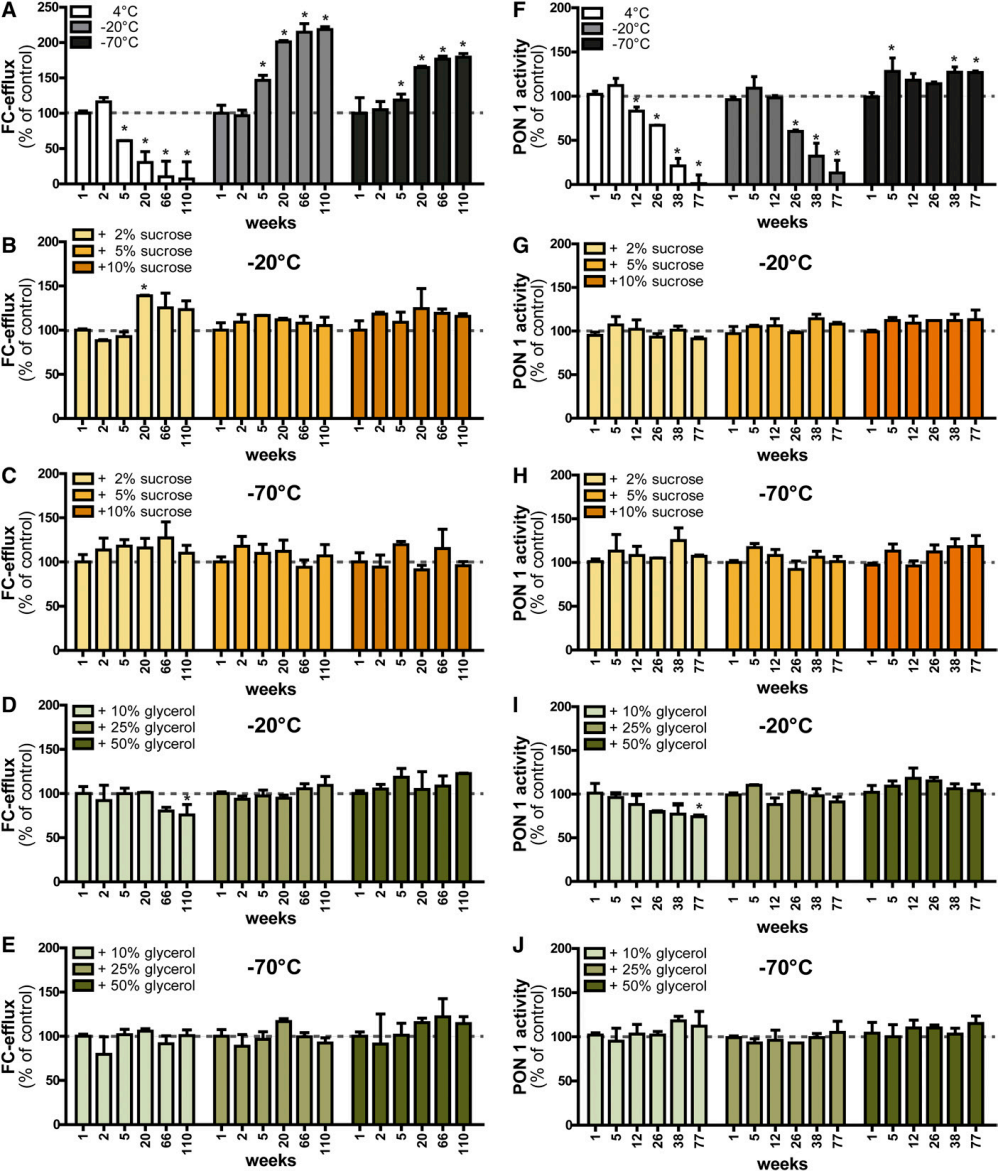
Functional analysis of cryopreserved HDL. HDL isolated by density gradient ultracentrifugation was stored at −20°C or −70°C in the presence or absence of the cryoprotectants, sucrose (2–10%) or glycerol (10–50%), for up to 110 weeks. Samples from four healthy donors were pooled and aliquots were analyzed for their ability to promote cholesterol efflux (A–E) and for their HDL-associated paraoxonase 1 activity (F–J) at indicated time points. The dashed lines indicate the control value measured right after the isolation of HDL and were used for normalization to 100%. Results represent the combined analysis of two independent experiments performed in triplicate. *P < 0.05 versus control.

We next tested the functional properties of frozen HDL, including cholesterol efflux capacity and arylesterase activity of HDL-associated paraoxonase. We observed that, depending on the storage condition of HDL, the cholesterol efflux capacity changed significantly (**Fig. 4A**). At 4°C, cholesterol efflux capacity was almost completely abolished at 110 weeks (Fig. 4A). At −20°C and −70°C, the efflux capacity increased up to 2-fold starting around week 5. Importantly, the efflux capacity remained constant upon the addition of at least 5% sucrose or 25% glycerol at −20°C (Fig. 4). The addition of the lower concentrations of sucrose (2%) and glycerol (10%) were not sufficient to completely maintain the efflux potential when stored at −20°C (Fig. 4B, D). At −70°C, the addition of 10% glycerol was sufficient to preserve the cholesterol efflux capability of HDL (Fig. 4E). Paraoxonase-1-mediated arylesterase activity was also affected depending on the storage condition. At 4°C and −20°C, arylesterase activity declined significantly until week 77 (Fig. 4F). Notably, HDL stored at −70°C showed significantly increased arylesterase activity. Addition of at least 5% sucrose or 25% glycerol as cryoprotectants was able to preserve arylesterase activity completely (Fig. 4F–J). Overall, these functional results were in good agreement with the observations from the structural analysis, indicating that structural changes directly translate into functional alterations. Importantly, the cryoprotectants alone did not interfere with cholesterol efflux measurements or arylesterase measurements (supplemental Fig. S2). Therefore, our results indicated that it is possible to use HDL with added sucrose or glycerol directly without the need of removal prior to experimentation, at least for the functional properties tested in this study.

**Fig. 5.**
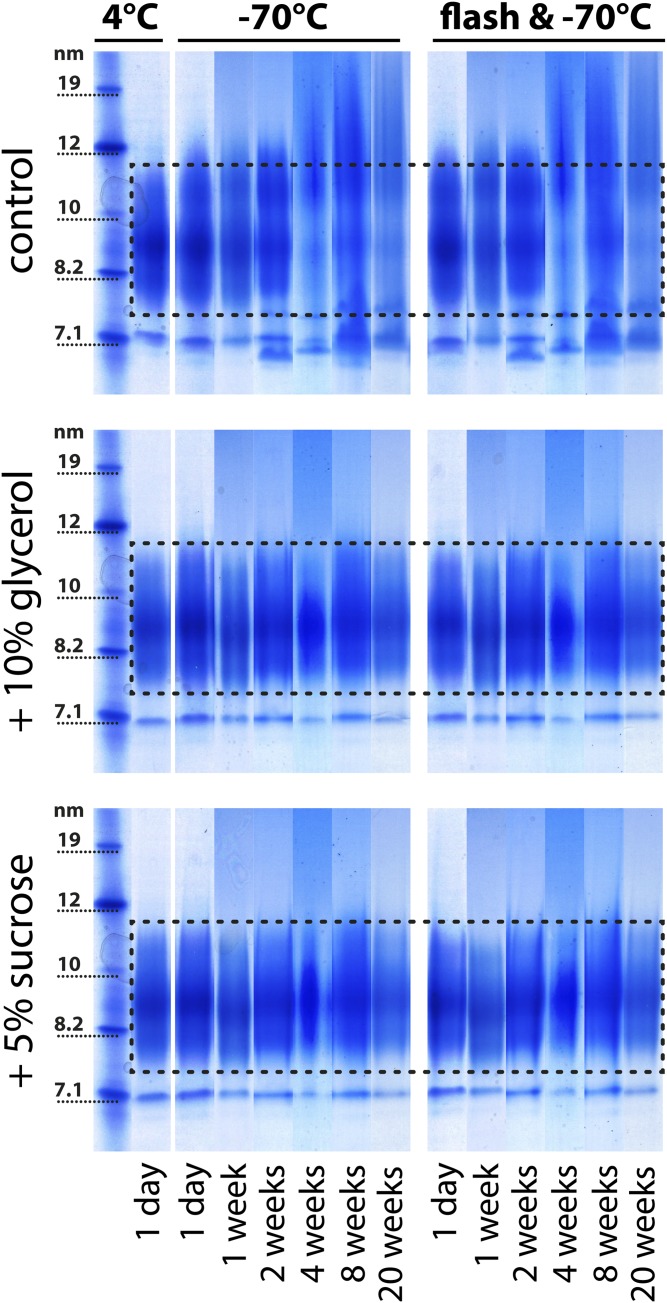
Flash-freezing of HDL. HDL was isolated from the serum of four healthy donors by density gradient ultracentrifugation and pooled for electrophoresis analysis. HDL with or without the cryoprotectants (10% glycerol or 5% sucrose) was either frozen directly at −70°C or flash-frozen with liquid nitrogen and subsequently stored at −70°C. Aliquots were analyzed at different time points and stained with Coomassie brilliant blue for protein. The dashed lines indicate the typical size range of HDL_2_ and HDL_3_ for the specific preparation ranging from about 7.5 to 11.5 nm. To be able to illustrate structural changes of HDL over time, we have placed the analyzed time points from each sample next to each other. The samples indicated with 4°C are baseline controls, which have been analyzed directly after isolation without freezing and after the addition of 10% glycerol or 5% sucrose. Each storage experiment was repeated three times and the results from one representative experiment are shown.

**Fig. 6.**
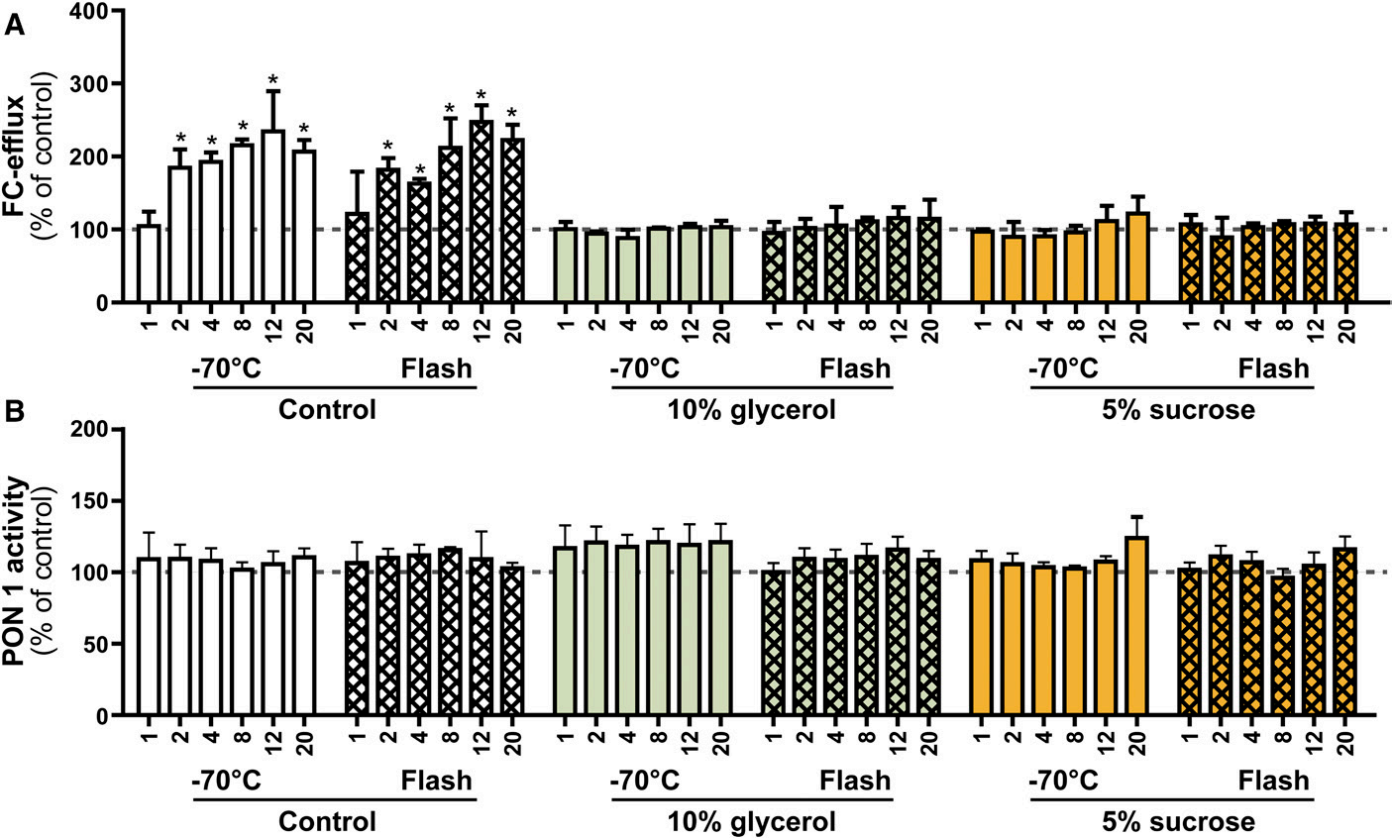
Functional analysis of flash-frozen HDL. HDL was isolated from the serum of four healthy donors by density gradient ultracentrifugation and pooled for functional analysis. HDL in the presence or absence of cryoprotectants (10% glycerol or 5% sucrose) was either frozen directly at −70°C or flash-frozen with liquid nitrogen and subsequently stored at −70°C. Aliquots were analyzed for their ability to promote cholesterol efflux (A) or for their HDL associated paraoxonase 1 activity (B). The dashed lines indicate the control value measured right after the isolation of HDL and were used for normalization to 100%. The labeling on the x axis indicates weeks of storage. Results represent the combined analysis of two independent experiments performed in triplicate. *P < 0.05 versus control.

Flash-freezing is a common practice for preserving biological material, especially tissue (28). So far, no data are available on whether flash-freezing with liquid nitrogen is suitable for isolated HDL. We tested the impact of flash-freezing prior to freezing at −70°C in the presence or absence of cryoprotectants. Our data indicated that flash-freezing alone does not prevent structural damage to HDL (**Fig. 5**). There was no difference evident on stained native gels between samples which had been frozen at −70°C directly and samples which had been flash-frozen before (Fig. 5), indicating that flash-freezing is not protective. Structural changes were completely prevented only when HDL was stored frozen in the presence of cryoprotectants (Fig. 5). Furthermore, we tested cholesterol efflux capability and arylesterase activity of flash-frozen HDL. Similar to the structural analysis, we observed that flash-freezing did not prevent changes in HDL functionality, only the addition of cryoprotectants maintained the metrics of HDL function (**Fig. 6**).

Prompted by these results, we studied recent HDL research articles to get insight about which storage strategies are commonly used. For that purpose, we reviewed the latest 100 research articles available in the PubMed database using the search terms “HDL ultracentrifugation” and “HDL proteome” (supplemental Table S1). Our analysis showed that, out of 100 articles, only 34 articles provided information regarding sample storage. In 11 publications, HDL was stored at 4°C and, in 23 publications, HDL was stored at −70/−80°C, while 64 publications did not provide any information regarding sample storage (**Table 1**). Most importantly, cryopreservation and/or flash-freezing were not described in any of the reviewed articles, providing clear evidence that cryopreservation is not a common practice. This review of the literature clearly suggests that there is not enough attention paid to the storage conditions of HDL.

**TABLE 1.** Description of storage conditions given in the 100 most recently published scientific articles using isolated HDL

Storage Condition	Number of Articles
4°C	13
−70/−80°C	21
Cryoprotectants	0
Flash-freezing	0
Not indicated	66

Articles were found via a PubMed search with the search terms “HDL ultracentrifugation” and “HDL proteome.” A full list of articles with authors, title, journal, and publication year is provided supplemental Table S1.

## DISCUSSION

It has been known for a long time that storage of biological material at freezing temperatures and subsequent thawing can cause significant damage (29). The use of cryoprotectants, such as glycerol, dimethylsulfoxid, sucrose, or others, has significantly improved the storage capabilities of biological material in experimental medicine (30, 31).

Lipoproteins are complex particles comprised of proteins and lipids. This complexity makes lipoproteins an interesting, but difficult to handle, study object. Often, too little attention is paid to sample storage. This is confirmed by our analysis of recently published articles, which indicated that, in 64% of publications, no information is provided on how HDL was stored. In this study, we investigated alterations in the physical and functional characteristics of HDL due to short- and long-term freezing using native gel electrophoresis in combination with cellular and enzymatic assays. All methods confirmed that HDL frozen in the presence of >5% sucrose or >25% glycerol remained structurally and functionally intact for at least 2 years and potentially for much longer periods of time. Isolated HDL stored at −20°C and −70°C without the use of cryoprotectants, showed significantly altered structure and dramatically altered function after short periods of time.

Alternatively, HDL can be stored at 4°C and our data indicate that it is stable for about 2 weeks (Fig. 1, supplemental Fig. S1). A recent report studied the effect of short-term freezing (48 h) on HDL functionality (32). The study used isolated HDL_2_ and it was noticed that the protein concentration of HDL decreased by 10% during the freeze-thaw process, which suggests particle damage during this period. However, functional analysis indicated that no significant changes in cholesterol efflux and the inhibitory activity of HDL on CD11b expression on monocytes occurred within the first 48 h of storage (32). This is in line with our observation that initial damage to HDL starts between week 1 and week 4 of storage, dependent on the individual isolated HDL (Fig. 2). However, because we used an optical method to analyze changes in HDL structure, small changes in HDL composition might occur much earlier. Similar observations were made when freezing studies with low density lipoprotein were performed, which have shown that freezing low density lipoprotein at −70°C in the absence of cryoprotectants resulted in fusion and aggregation of particles (33). These structural changes were accompanied by a 3- to 10-fold increase in binding to fibroblasts and their subsequent uptake (33). Flash freezing with liquid nitrogen is a commonly used practice to preserve biological material, especially tissue. Our data suggest that flash-freezing does not prevent storage-induced damage to HDL. We also did not find a benefit of a combination of flash-freezing and cryoprotectants. This is in line with observations that fast freezing of biological material is more harmful than slow and steady freezing (28, 34).

Some reports have described that the measurement of HDL-cholesterol levels in whole serum/plasma by precipitation can be significantly affected by storage conditions. Stored at −20°C, HDL-cholesterol concentration decreased by 4.8% per year and HDL_3_-cholesterol decreased by 6.9% per year (35). Thus, long-term storage of plasma at −20°C does not seem to be appropriate for subsequent quantification of HDL-cholesterol and its subclasses after precipitation (25). Storage at −70°C was preferable, but also led to a decrease in HDL protein content (36), which is in good agreement with our results from structural analysis. In a recent study, the impact of storage at −20°C on the distribution of apoC-III and apoE within HDL was investigated (37). In this study, HDL was isolated from fresh serum samples or from serum samples that had been stored for 2 weeks at −20°C. The authors observed that HDL isolated from frozen material contained a significantly higher amount of apoC-III and apoE, while less apoC-III and apoE were recovered in apoB-containing lipoproteins (37). These data demonstrate that storage can cause a time- and temperature-dependent redistribution of proteins between lipoproteins. To minimize any alteration in HDL structure and function, it is advisable to isolate HDL as soon as possible after serum sampling. However, further studies are needed to determine what delay in HDL isolation is acceptable to get intact HDL preparations.

Native gel analysis within our study indicated that prolonged frozen storage promotes dissociation of proteins from HDL and large protein-poor particles are formed, presumably by particle fusion. Immunoblot analysis identified that mainly apoA-I is shed from HDL when stored frozen in the absence of cryoprotectants, while other major HDL proteins, such as apoA-II, apoC-I, and SAA, appear to dissociate from mature HDL to a much lower extent (Fig. 3). Interestingly, similar observations have been made when thermal stability of HDL was tested (38, 39). These studies reported two distinct populations of apolipoproteins on HDL: one readily exchangeable and the other more strongly associated with the particle (40). In thermal stability experiments, HDL fusion involved the dissociation of apoA-I, while apoA-II that inserts deeper into the lipid layer, remained HDL particle associated. ApoA-I dissociation appears to produce transient hydrophobic packing defects on the lipoprotein surface, promoting lipoprotein fusion and aggregation (41). Therefore, one may assume that dissociation of apoA-I during prolonged storage of HDL in the frozen state promotes particle fusion. Further thermodynamic studies are needed to get deeper insights. These studies will be complex and very hard to perform, given that apoA-I shedding in the frozen state is very slow.

Proteins lost from intact HDL_2_ and HDL_3_ appear to accumulate in the size range of 7.5 nm, clearly visible on native gels (Fig. 1). This observation suggests that lipid-poor forms of HDL are formed, which might resemble preβ-HDL in structure and function. This might be of particular relevance for the cholesterol efflux experiments that were performed. In these experiments, we used a macrophage cell line stimulated with cyclic AMP to induce ABCA1 expression, an established method for quantifying HDL function (27). This efflux system is sensitive to changes in the amount of poorly lipidated forms of HDL (42) because lipid-poor HDL is the main acceptor of cholesterol effluxed from ABCA1. Therefore, the marked increase in HDL-cholesterol efflux capacity after an extended period of sample storage is most likely attributable to the formation of lipid-poor HDL particles.

Paraoxonase 1 localization on HDL is critically important for its activity, by stabilizing the enzyme and providing an optimal environment for the interaction with its physiological substrates (43). Paraoxonase 1 binds to HDL through the interaction of its hydrophobic N terminus with phospholipids (44). Structural models imply that HDL anchoring modifies the active site of paraoxonase 1 and significantly enhances its enzymatic activities (43). The decline in HDL-associated paraoxonase activity that we observed during extended storage periods of HDL is potentially caused by the disruption of HDL-paraoxonase 1 interactions. This is likely a result from the loss of proteins from mature HDL_2_ and HDL_3_ and the accumulation of proteins in the lipid-poor HDL size of about 7.5 nm (Fig. 1).

We have provided compelling evidence that HDL isolated by ultracentrifugation changes its structure and function upon storage at −20°C or −70°C. However, based on our data, we cannot judge whether different isolation methods could have an impact on the storage ability of HDL. Recent reports have shown that the protein composition can be altered by the isolation procedure, which could potentially change the ability to store HDL at subzero temperatures (45). However, these observations warrant further investigation.

In clinical practice, it is generally recommended that plasma samples that require measurements of HDL-cholesterol and HDL subfractions by precipitation be stored refrigerated at 0–4°C and analyzed within 24 h of blood collection. However, no such guideline exists for isolated HDL and its subsequent use for structural and functional analysis. Based on our data, we recommend that HDL should be isolated from serum/plasma as soon as possible to allow a reliable and comparable characterization of HDL structure and function. The use of cryoprotectants will allow the long-term storage of samples and subsequent analysis of a variety of structural and functional parameters.

We are confident that our study can have a profound impact on the HDL research community. Our data will raise the awareness that HDL is a complex particle requiring special attention when stored and, thereby, improving the reproducibility and quality of research data obtained. In particular, when HDL is isolated from controls and patients at different time points (which is very often the case), storage of HDL in the absence of cryoprotectants might profoundly affect results, given that HDL-cholesterol efflux capacity might increase 2-fold over time. We hope that addition of cryoprotectants to isolated HDL samples before storage will make biochemical and clinical HDL research studies more reproducible and comparable.

## Supplementary Material

Supplemental Data
