# Supplementary material for: HDL structure and function is profoundly affected when stored frozen in the absence of cryoprotectants
Source: J Lipid Res. 2017 Sep 11;58(11):2220–8. doi: 10.1194/jlr.D075366 (PMC5665661; doi:10.1194/jlr.D075366)
Supplement: Supplemental Data [file supp_58_11_2220__index.html]

HDL structure and function is profoundly affected when stored frozen in the absence of cryoprotectants — HDL structure and function is profoundly affected when stored frozen in the absence of cryoprotectants — Supplemental Data 

# HDL structure and function is profoundly affected when stored frozen in the absence of cryoprotectants

## Supplemental Data

- Supplemental material (.pdf, 582 KB) - Supplemental material
