# Supplementary material for: HDL structure and function is profoundly affected when stored frozen in the absence of cryoprotectants
Source: J Lipid Res. 2017 Sep 11;58(11):2220–8. doi: 10.1194/jlr.D075366 (PMC5665661; doi:10.1194/jlr.D075366)
Supplement: Supplemental Data [file 10.1194_D075366_jlr.D075366-1.pdf]

SUPPLEMENTAL MATERIAL

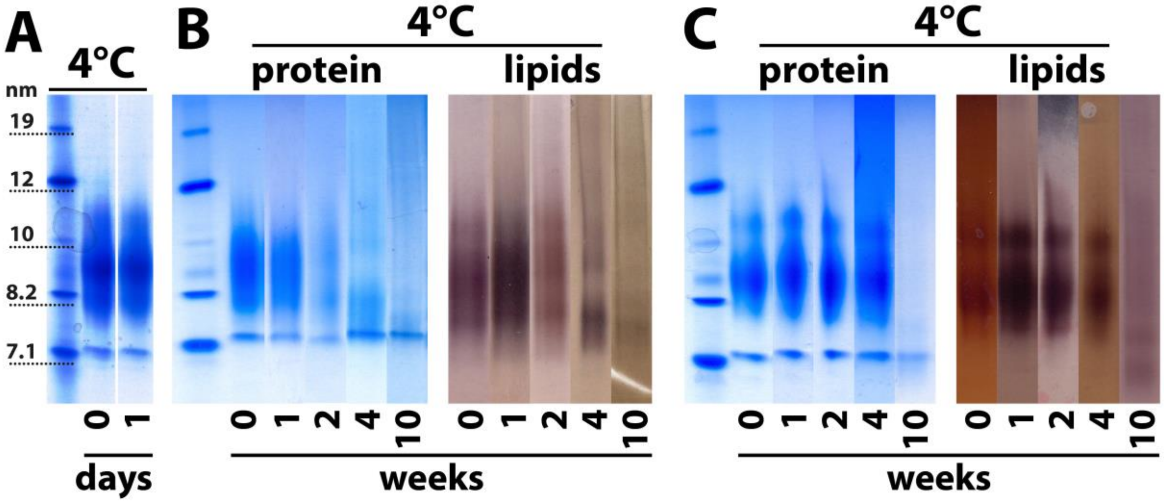

Supplemental Figure 1: Impact of storage of isolated HDL at 4°C. HDL was isolated from 4 healthy donors by ultracentrifugation and pooled for analysis as described in the methods section. HDL was analyzed via native gel electrophoresis either right after isolation or stored at 4°C for the indicated periods. Gels were stained with Coomassie brilliant blue for protein or with sudan black for lipids. The HDL samples came from different gels and therefore have been cut and assembled for illustration. The HDL samples shown in panel A, B and C were from three different preparations.

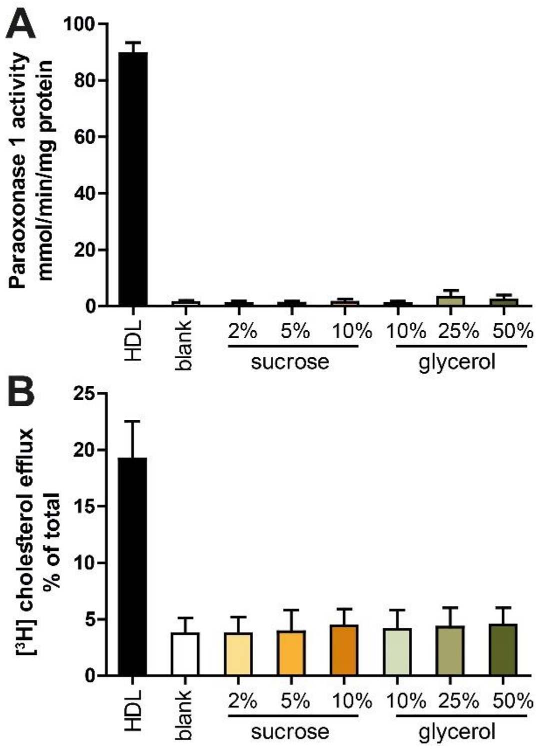

Supplemental Figure 2: Impact of cryoprotectants on functional measurements of HDL. HDL or the cryoprotectants sucrose (2-10%) or glycerol (10-50%) were tested for the influence on (A) HDL associated paraoxonase 1 activity or (B) cholesterol efflux measurements. Results represent the combined analysis of two independent experiments performed in triplicates.

Supplemental table 1: Description of storage conditions given in recently published scientific articles using isolated HDL.

List was derived from Pubmed using the search terms “HDL ultracentrifugation” and “HDL proteome”. The first 100 papers using ultracentrifugation for HDL preparation from human material were selected. The method section of each paper was searched for a description of how isolated HDL was stored after isolation and during experimental investigation. “Not indicated” in the storage column identifies papers which did not provide any information about how HDL samples were stored.

Reference	Title	YEAR	Journal	Storage
Frej et al.	A Shift in ApoM/S1P between HDL-particles in Women with Type 1 diabetes is associated with impaired anti-inflammatory effects of the apoM/S1P-complex	2017	ATVB	not indicated
Ljunggren et al.	Alterations in high-density lipoprotein proteome and function associated with persistent organic pollutants	2017	Environ Int.	not indicated
Maki et al.	Corn oil intake favorably impacts lipoprotein cholesterol, apolipoprotein and lipoprotein particle levels compared with extra-virgin olive oil.	2017	Eur J Clin Nutr.	not indicated
Norimatsu et al.	Significance of the percentage of cholesterol efflux capacity and total cholesterol efflux capacity in patients with or without coronary artery disease.	2017	Heart Vessels	not indicated
Paavola et al.	Impaired HDL2-mediated cholesterol efflux is associated with metabolic syndrome in families with early onset coronary heart disease and low HDL-cholesterol level	2017	Plos One	-70°C
Abdullah et al.	Amyloid-β Reduces Exosome Release from Astrocytes by Enhancing JNK Phosphorylation.	2016	J Alzheimers Dis	not indicated
Burillo et al.	Quantitative HDL Proteomics Identifies Peroxiredoxin-6 as a Biomarker of Human Abdominal Aortic Aneurysm.	2016	Scientific Reports	not indicated
Cubedo et al.	ApoL1 levels in high density lipoprotein and cardiovascular event presentation in patients with familial hypercholesterolemia.	2016	J Lipid Res.	-80°C
Delbosc et al.	High-density lipoprotein therapy inhibits Porphyromonas gingivalis-induced abdominal aortic aneurysm progression.	2016	Thromb Haemost.	not indicated
Godzien et al.	A Single In-Vial Dual Extraction Strategy for the Simultaneous Lipidomics and Proteomics Analysis of HDL and LDL Fractions.	2016	J Proteome Res.	not indicated

Hansel et al.	Lifestyle intervention enhances high-density lipoprotein function among patients with metabolic syndrome only at normal low-density Q1 lipoprotein cholesterol plasma levels	2016	J Clin Lipidol.	not indicated
Henderson et al.	Isolating and Quantifying Plasma HDL Proteins by Sequential Density Gradient Ultracentrifugation and Targeted Proteomics.	2016	Methods Mol Biol.	4°C
Holzer et al.	Refined purification strategy for reliable proteomic profiling of HDL2/3: Impact on proteomic complexity.	2016	Scientific Reports	-70°C
Huang et al.	HDL of patients with breast cancer complicated with type 2 diabetes mellitus promotes cancer cells adhesion to vascular endothelium via ICAM-1 and VCAM-1 upregulation.	2016	Breast Cancer Res Treat	4°C
Hughes et al.	Lipoprotein composition in patients with type 1 diabetes mellitus: Impact of lipases and adipokines.	2016	J Diabetes Complic.	not indicated
Jansen et al.	In silico modeling of the dynamics of low density lipoprotein composition via a single plasma sample.	2016	J Lipid Res.	not indicated
Manjunatha et al.	Functional and proteomic alterations of plasma high density lipoproteins in type 1 diabetes mellitus.	2016	Metabolism	not indicated
McGowan et al.	Postprandial Studies Uncover Differing Effects on HDL Particles of Overt and Subclinical Hypothyroidism.	2016	Thyroid	not indicated
Michell et al.	Isolation of High-density Lipoproteins for Non-coding Small RNA Quantification	2016	J Vis Exp.	-70°C
Oberbach et al.	Proteome profiles of HDL particles of patients with chronic heart failure are associated with immune response and also include bacteria proteins.	2016	Clin Chim Acta.	not indicated
Ortiz-Munoz et al.	Dysfunctional HDL in acute stroke.	2016	Atherosclerosis	not indicated
Pan et al.	A novel anti-inflammatory mechanism of high density lipoprotein through up-regulating annexin A1 in vascular endothelial cells.	2016	BBA	4°C
Proudfoot et al.	F2-Isoprostanes in HDL are bound to neutral lipids and phospholipids.	2016	Free Radic Res.	4°C
Ronsein et al.	Targeted Proteomics Identifies PON1 and Apolipoprotein Cs as Potential Risk Factors for Hypoalphalipoproteinemia in Diabetic Subjects Treated with Fenofibrate and Rosiglitazone.	2016	Mol Cell Proteomics.	not indicated
Trieb et al.	Liver disease alters high-density lipoprotein composition, metabolism and function.	2016	BBA	not indicated
Weckerle et al.	Characterization of circulating APOL1 protein complexes in African Americans.	2016	J Lipid Res.	not indicated

Zhao et al.	Different relationship between ANGPTL3 and HDL components in female non-diabetic subjects and type-2 diabetic patients.	2016	Cardiovasc Diabetol.	not indicated
Calderon et al.	Elevated Lipoprotein Lipase Activity Does Not Account for the Association Between Adiponectin and HDL in Type 1 Diabetes	2015	J Clin Endocrinol Metab.	not indicated
Curcic et al.	Neutrophil effector responses are suppressed by secretory phospholipase A2 modified HDL.	2015	BBA	-70°C
Fernandez et al.	Re-evaluation of the anticoagulant properties of high-density lipoprotein-brief report.	2015	ATVB	not indicated
Guardiola et al.	Lipoprotein-induced phenoloxidase-activity in tarantula hemocyanin.	2015	J Clin Lipidol.	not indicated
Hescot et al.	Lipoprotein-Free Mitotane Exerts High Cytotoxic Activity in Adrenocortical Carcinoma.	2015	J Clin Endocrinol Metab.	not indicated
Holzer et al.	Dialysis Modalities and HDL Composition and Function.	2015	JASN	-70°C
Homma et al.	Changes in ultracentrifugally separated plasma lipoprotein subfractions in patients with polygenic hypercholesterolemia, familial combined hyperlipoproteinemia, and familial….	2015	J Clin Lipidol.	not indicated
Kopecky et al.	Quantification of HDL proteins, cardiac events, and mortality in patients with type 2 diabetes on hemodialysis.	2015	CJASN	-80°C
Kopecky et al.	Restoration of renal function does not correct impairment of uremic HDL properties.	2015	JASN	-80°C
Krishnan et al.	Combined High-Density Lipoprotein Proteomic and Glycomic Profiles in Patients at Risk for Coronary Artery Disease.	2015	J Proteome Res.	not indicated
Kulanuwat et al.	Prevalence of plasma small dense LDL is increased in obesity in a Thai population.	2015	Lipids Health Dis.	not indicated
Ljunggren et al.	Lipoprotein profiles in human heterozygote carriers of a functional mutation P297S in scavenger receptor class B1.	2015	BBA	not indicated
Manita et al.	A rapid anion-exchange chromatography for measurement of cholesterol concentrations in five lipoprotein classes and estimation of lipoprotein profiles in male volunteers without…	2015	Ann Clin Biochem.	not indicated
Marsillach et al.	Paraoxonase-3 is depleted from the high density lipoproteins of autoimmune diseasepatients with subclinical atherosclerosis	2015	J Proteome Res.	not indicated
Martin et al.	HDL cholesterol subclasses, myocardial infarction, and mortality in secondary prevention: the Lipoprotein Investigators Collaborative.	2015	Eur Heart J.	not indicated

McEneny et al.	Serum- and HDL3-serum amyloid A and HDL3-LCAT activity are influenced by increased CVD-burden	2015	Atherosclerosis	-80°C
McEneny et al.	A Cross-Sectional Study Demonstrating Increased SAA Related Inflammation in HDL from Subjects with Type 1 Diabetes Mellitus and How this Association Was Augmented….	2015	J Diabetes Res.	not indicated
Merono et al.	Metabolic alterations, HFE gene mutations and atherogenic lipoprotein modifications in patients with primary iron overload.	2015	Clin Sci (Lond).	4°C
Munroe et al.	Excessive centrifugal fields damage high density lipoprotein.	2015	J Lipid Res.	not indicated
Niculescu et al.	MiR-486 and miR-92a Identified in Circulating HDL Discriminate between Stable and Vulnerable Coronary Artery Disease Patients.	2015	PLoS One.	-80°C
O'Neill et al.	Structural and functional changes in HDL with low grade and chronic inflammation.	2015	Int J Cardiol.	not indicated
Pedret et al.	Impact of Virgin Olive Oil and Phenol-Enriched Virgin Olive Oils on the HDL Proteome in Hypercholesterolemic Subjects (VOHF Study).	2015	PLoS One.	-80°C
Rached et al.	Defective functionality of small, dense HDL3 subpopulations in ST segment elevation myocardial infarction: Relevance of enrichment in LPC, phosphatidic acid and SAA.	2015	BBA	4°C
Ronsein et al.	Parallel reaction monitoring (PRM) and selected reaction monitoring (SRM) exhibit comparable linearity, dynamic range and precision for targeted quantitative HDL proteomics.	2015	J Proteomics	not indicated
Rull et al.	Increased concentration of clusterin/apolipoprotein J (apoJ) in hyperlipemic serum is paradoxically associated with decreased apoJ content in lipoproteins	2015	Atherosclerosis	not indicated
Salminen et al.	Matrix metalloproteinase 8 degrades apolipoprotein A-I and reduces its cholesterol efflux capacity.	2015	FASEB J.	not indicated
Sanchez et al.	Low concentrations of phospholipids and plasma HDL cholesterol subclasses in asymptomatic subjects with high coronary calcium scores	2015	Atherosclerosis	not indicated
Sattler et al.	Defects of High-Density Lipoproteins in Coronary Artery Disease Caused by Low Sphingosine-1-Phosphate Content: Correction by Sphingosine-1-Phosphate-Loading.	2015	JACC	not indicated
Serna et al.	Quantitative lipidomic analysis of plasma and plasma lipoproteins using MALDI-TOF mass spectrometry.	2015	Chem Phys Lipids.	not indicated
Shao et al.	A Cluster of Proteins Implicated in Kidney Disease Is Increased in High-Density Lipoprotein Isolated from Hemodialysis Subjects.	2015	J Proteome Res.	not indicated
Shrestha et al.	Identification of molecular species of oxidized triglyceride in plasma and its distribution in lipoproteins.	2015	Clin Chem Lab Med.	-80°C

Siegel et al.	HIV infection induces structural and functional changes in high density lipoproteins.	2015	Atherosclerosis	not indicated
Sreckovic et al.	Gestational diabetes mellitus modulates neonatal high-density lipoprotein composition and its functional heterogeneity.	2015	BBA	4°C
Vaisar et al.	Inflammatory remodeling of the HDL proteome impairs cholesterol efflux capacity.	2015	J Lipid Res.	not indicated
Xiong et al.	The association of HDL-apoCIII with coronary heart disease and the effect of statin treatment on it.	2015	Lipids Health Dis.	not indicated
Daniels et al.	A randomised controlled trial of increasing fruit and vegetable intake and how this influences the carotenoid concentration and activities of PON-1 and LCAT in HDL…	2014	Cardiovasc Diabetol.	-80°C
DiDonato	Site-specific nitration of apolipoprotein A-I at tyrosine 166 is both abundant within human atherosclerotic plaque and dysfunctional.	2014	J Biol Chem.	not indicated
Gidwani et al.	Polycystic ovary syndrome influences the level of serum amyloid A and activity of phospholipid transfer protein in HDL_2 and HDL_3.	2014	Hum Reprod.	not indicated
Huang et al.	Glycomic analysis of high density lipoprotein shows a highly sialylated particle.	2014	J Proteome Res.	-80°C
Hutchins et al.	Quantification of HDL particle concentration by calibrated ion mobility analysis.	2014	Clin Chem.	not indicated
Ito et al.	Development of a homogeneous assay for measurement of high-density lipoprotein-subclass cholesterol.	2014	Clin Chim Acta.	not indicated
Ljunggren et al.	ApoA-I mutations, L202P and K131del, in HDL from heterozygotes with low HDL-C.	2014	Proteomics Clin Appl.	not indicated
Nadeem et al.	Serum amyloid A-related inflammation is lowered by increased fruit and vegetable intake, while high-sensitive C-reactive protein, IL-6 and E-selectin remain unresponsive.	2014	Br J Nutr.	-75°C
Nakamura	LDL cholesterol performance of beta quantification reference measurement procedure.	2014	Clin Chim Acta.	not indicated
Rached et al.	Defective functionality of HDL particles in familial apoA-I deficiency: relevance of alterations in HDL lipidome and proteome.	2014	J Lipid Res.	4°C
Richard et al.	Effect of an isoenergetic traditional Mediterranean diet on the high-density lipoprotein proteome in men with the metabolic syndrome.	2014	J Nutrigenet Nutrigenomics.	not indicated
Rosso et al.	Altered lipidome and antioxidative activity of small, dense HDL in normolipidemic rheumatoid arthritis: relevance of inflammation.	2014	Atherosclerosis	4°C

Schenk et al.	Lipoprotein-induced phenoloxidase-activity in tarantula hemocyanin.	2014	BBA	4°C
Sini et al.	Evidence for an exclusive association of matrix metalloproteinase-9 with dysfunctional high-density lipoprotein: a novel finding.	2014	Atherosclerosis	not indicated
Tan et al.	Acute coronary syndrome remodels the protein cargo and functions of high-density lipoprotein subfractions.	2014	Plos One	-70°C
Williams et al.	Comparison of four methods of analysis of lipoprotein particle subfractions for their association with angiographic progression of coronary artery disease.	2014	Atherosclerosis	-80°C
Yan et al.	A pro-atherogenic HDL profile in coronary heart disease patients: an iTRAQ labelling-based proteomic approach.	2014	Plos One	-80°C
Yassine et al.	The application of multiple reaction monitoring and multi-analyte profiling to HDL proteins.	2014	Lipids Health Dis.	not indicated
Yuana et al.	Co-isolation of extracellular vesicles and high-density lipoproteins using density gradient ultracentrifugation.	2014	J Extracell Vesicles.	not indicated
Zychlinski et al.	Absolute quantification of apolipoproteins and associated proteins on human plasma lipoproteins.	2014	J Proteomics.	not indicated
Adams et al.	Exercise training in patients with chronic heart failure promotes restoration of high-density lipoprotein functional properties.	2013	Circ Res.	4°C
Boshtam et al.	Serum paraoxonase 1 activity is associated with fatty acid composition of high density lipoprotein.	2013	Dis Markers.	-80°C
Gaal et al.	Characterization of a novel high-density lipoprotein antioxidant capacity assay and its application to high-density lipoprotein fractions.	2013	Clin Biochemistry	not indicated
Holzer et al.	Aging affects high-density lipoprotein composition and function.	2013	BBA	-70°C
Homma et al.	Skew of plasma low- and high-density lipoprotein distributions to less dense subfractions in normotriglyceridemic CKD patients on maintenance hemodialysis treatment.	2013	Nephron Clin Pract.	not indicated
Huang et al.	Myeloperoxidase, paraoxonase-1, and HDL form a functional ternary complex.	2013	JCI	not indicated
Lepeda et al.	Proteomic analysis of plasma-purified VLDL, LDL, and HDL fractions from atherosclerotic patients undergoing carotid endarterectomy…	2013	Oxid Med Cell Longev.	not indicated
Matsuo et al.	Impaired HDL function in obese adolescents: impact of lifestyle intervention and bariatric surgery.	2013	Obesity	not indicated

McEneny et al.	High-density lipoprotein subfractions display proatherogenic properties in overweight and obese children.	2013	Pediatr Res.	not indicated
McNeal et al.	Human HDL containing a novel apoC-I isoform induces smooth muscle cell apoptosis.	2013	Cardiovasc Res.	not indicated
Müller et al.	Correlation between endothelial function measured by finger plethysmography in children and HDL-mediated eNOS activation -- a preliminary study.	2013	Metabolism	not indicated
Nguyen et al.	Interactions of apolipoprotein A-I with high-density lipoprotein particles.	2013	Biochemistry	not indicated
Nguyen et al.	Secreted progranulin is a homodimer and is not a component of high density lipoproteins (HDL).	2013	J Biol Chem.	not indicated
Razavi et al.	Associations between high density lipoprotein mean particle size and serum paraoxonase-1 activity.	2013	J Res Med Sci.	not indicated
Riwanto et al.	Altered activation of endothelial anti- and proapoptotic pathways by HDL from patients with coronary artery disease: role of high-density lipoprotein-proteome remodeling.	2013	Circulation.	not indicated
Saha et al.	Stimulation of phagocyte adhesion to endothelial cells by modified VLDL and HDL requires scavenger receptor BI.	2013	Mol Cell Biochem.	not indicated
Sampaio et al.	Impaired antioxidant action of high density lipoprotein in patients with type 1 diabetes with normoalbuminuria and microalbuminuria.	2013	Diabetes Res Clin Pract.	-70°C
Segrest et al.	Volumetric determination of apolipoprotein stoichiometry of circulating HDL subspecies.	2013	J Lipid Res.	not indicated
Sreckovic et al.	Distinct composition of human fetal HDL attenuates its anti-oxidative capacity.	2013	BBA	4°C
Tong et al.	HDL of patients with type 2 diabetes mellitus upregulates cyclooxgenase-2 expression and prostacyclin I-2 release in endothelial cells…	2013	Cardiovasc Diabetol.	4°C
